# A concordance-based study to assess doctors’ and nurses’ mental models in Internal Medicine

**DOI:** 10.1371/journal.pone.0182608

**Published:** 2017-08-08

**Authors:** Katherine S. Blondon, K. C. Gary Chan, Virginie Muller-Juge, Stéphane Cullati, Patricia Hudelson, Fabienne Maître, Nu V. Vu, Georges L. Savoldelli, Mathieu R. Nendaz

**Affiliations:** 1 Division of General Internal Medicine, Department of General Internal Medicine, Geriatrics and Rehabilitation, University Hospitals of Geneva, Geneva, Switzerland; 2 Department of Biostatistics and Department of Health Services, University of Washington, Seattle, United States of America; 3 Unit of Development and Research in Medical Education (UDREM), Faculty of Medicine, University of Geneva, Geneva, Switzerland; 4 Quality of Care Service, University Hospitals of Geneva, Geneva, Switzerland; 5 Department of Community Medicine, Primary Care and Emergency Medicine, University Hospitals of Geneva, Geneva, Switzerland; 6 Division of Anaesthesiology, University Hospitals of Geneva, Geneva, Switzerland; Monash University, AUSTRALIA

## Abstract

Interprofessional collaboration between doctors and nurses is based on team mental models, in particular for each professional’s roles. Our objective was to identify factors influencing concordance on the expectations of doctors’ and nurses’ roles and responsibilities in an Internal Medicine ward. Using a dataset of 196 doctor-nurse pairs (14x14 = 196), we analyzed choices and prioritized management actions of 14 doctors and 14 nurses in six clinical nurse role scenarios, and in five doctor role scenarios (6 options per scenario). In logistic regression models with a non-nested correlation structure, we evaluated concordance among doctors and nurses, and adjusted for potential confounders (including prior experience in Internal Medicine, acuteness of case and gender). Concordance was associated with number of female professionals (adjusted OR 1.32, 95% CI 1.02 to 1.73), for acute situations (adjusted OR 2.02, 95% CI 1.13 to 3.62), and in doctor role scenarios (adjusted OR 2.19, 95% CI 1.32 to 3.65). Prior experience and country of training were not significant predictors of concordance. In conclusion, our concordance-based approach helped us identify areas of lower concordance in expected doctor-nurse roles and responsibilities, particularly in non-acute situations, which can be targeted by future interprofessional, educational interventions.

## Introduction

Inpatient hospital care requires close interprofessional collaboration between doctors and nurses. Effective collaboration can improve the quality and cost of care [1–5]. Good team collaboration requires having a team mental model among the team members [6, 7], which has been defined as “organized mental representations of the key elements within a team’s relevant environment that are shared across team members” [8]. Two contexts for team mental models have been described in team science literature: taskwork and teamwork [9]. The taskwork model captures the members’ understanding of resources, strategies, procedures and task contingencies. The teamwork model reflects the members’ perception of team member roles, responsibilities and norms, and of team structure, coordination and communication. Assessment of mental models is considered through 3 aspects, according to a conceptual framework summarized by DeChurch: [10] a) elicitation methods, or the technique to elicit the content in the model, b) structure representation or the associative networks of knowledge, which can be assessed with similarity ratings and c) the degree of sharedness, or degree of overlap between mental models in a team. Our study aimed at rapidly and feasibly assessing the degree of sharedness between the team, using scenarios that require clinical assessment, reasoning and notions of role representation and expectations. Teams rely on a “pattern of cognitive similarity to effectively retrieve and share information.” [11]. In fact, shared expectations and perceptions of professional roles lay the groundwork for communication and collaboration for effective team performance [12]. Lack of a shared underlying mental model of roles can therefore have potential repercussions on quality of care [7, 13] and patient safety [14, 15]. Important differences however have been shown between behaviors and role expectations among nurses and physicians [1, 16, 17]. Perception of poor collaboration with physicians is also associated with nurse's intentions to leave their job [18, 19].

As health professions education moves towards interprofessional education at both graduate and post-graduate levels [20, 21], there is a growing need to develop methods to assess underlying role expectations and perceptions, as groundwork for teamwork improvement. Addressing misunderstandings can allow programs to improve communication and collaboration. For this paper, we will use the term “role” to describe the health professional’s role, responsibilities and activities.

In a previous paper on interprofessional collaboration between doctors and nurses in General Internal Medicine wards, we explored and compared role expectations and perceptions among these professions [22]. Using a mixed methods approach with individual interviews and a questionnaire, we studied how doctors and nurses viewed their own role and the role of the other profession. Results indicated only moderate correlation between doctors’ and nurses’ expectations and intended actions, suggesting the need for better clarity regarding doctors’ and nurses’ roles, particularly regarding nurse autonomy: recognizing and anticipating patient problems and involvement in decision-making [22].

In this paper, our objective was to assess factors associated with a concordant team mental model, based on doctor and nurse teams in an Internal Medicine ward, using a clinical case-based questionnaire. Our analysis focuses on factors related to the working environment (undergraduate training and current work place), the clinical case (acute or not) and team (role expectations and perceptions). We hypothesized that greater prior experience in an internal medicine environment would improve concordance, through exposure to a common environment with similar local practices. Studies in team science have shown that sharing a team mental model can help anticipate the other members’ responses, with improved coordination and communication [9, 23]. Likewise, we also hypothesized that experience acquired during graduate training in Switzerland would be associated with higher concordance among doctors and nurses compared to graduate training in other countries such as France, due to differences in team organization, procedures and environmental conditions. Prior literature has shown that team mental models can enhance coordination and effectiveness in urgent contexts. We therefore hypothesized that acute cases would have higher concordance between doctors and nurses compared with non-acute cases. Acute cases may have more clearly identified management roles than non-acute cases, as many algorithms for acute situations have already been established. Finally, we hypothesized that concordance would be higher regarding doctors’ roles than nurses’ roles, based on our prior qualitative findings [22].

## Methods

We used the materials collected for the mixed-approach study [22] to conduct a secondary analysis of the questionnaire dataset. The overall project was approved by the research ethics committee of the University Hospitals of Geneva, which waived a complete review.

### Participants and setting

The study was conducted with a convenience sample of 14 doctors and 14 nurses recruited from the Division of General Internal Medicine at the Geneva University Hospitals, Switzerland, a 1800-bed public hospital. This division encompasses 130 beds across nine acute-care wards. The participants were residents from a 5-year post-graduate training program and staff nurses actively working on the Internal Medicine ward.

### Data collection and analysis

Participants were asked to fill out a questionnaire with 11 clinical scenarios. In six of these, participants were asked what action the nurse should take, and in five they were asked what action the doctor should take (Table 1). Depending on the profession of the participant, the questions were worded as “Indicate how *you* would deal with the situation” or “Indicate how you think the doctor/nurse should deal with the situation”. Participants were asked to select at least one action (from a list of six possible actions) for each scenario, but could select two or more ranked actions if desired. These six possible actions reflect role perceptions and role expectations, as well as the degree of perceived urgency.

**Table 1:** Example of a scenario (translated from French).

Question for the doctor	Question for the nurse
A 65 year-old patient was admitted for myocardial infarction 4 days ago. He’d been feeling better until this morning: he rings the bell and says that he has chest pain. He is sweating and uncomfortable. Blood pressure 140/70 mmHg, irregular pulse of 120/min, and respiratory rate 24/min.	A 65 year-old patient was admitted for myocardial infarction 4 days ago. He’d been feeling better until this morning: he rings the bell and says that he has chest pain. He is sweating and uncomfortable. Blood pressure 140/70 mmHg, irregular pulse of 120/min, and respiratory rate 24/min.
How should the nurse handle this situation? She/he should: A. Call the medical emergency team B. Page you C. Page the attending D. Call the head nurse E. Wait for rounds F. Deal with it her/himself: describe the expected actions	How would you handle this situation? You would: A. Call the medical emergency team B. Page the resident C. Page the attending D. Call the head nurse E. Wait for rounds F. Deal with it yourself: describe your actions

Each scenario required clinical reasoning, and integrated the notions of doctor and nurse roles and expectations in a clinical setting, and goes beyond the application of a procedure. The assessment of urgency exemplified in Table 1 is a key aspect of the mental model, and will affect the plan of action chosen.

We built logistic regression models using concordance between doctor and nurse pairs as a primary outcome. Pairs were the unit of analysis and were built by theoretically combining the 14 doctors with the 14 nurses (14*14 = 196 pairs). For every question within a doctor-nurse pair, we created a binary variable for concordance between each doctor and each nurse (concordance versus no concordance). Concordance was defined by either (1) doctor-nurse agreement on the initial response (top choice) of one type of professional in a specific situation, or (2) agreement on at least one of the top two choices. We proceeded initially using the top choice for concordance, and then broadened the analysis to the first two choices given for each clinical scenario. For these clinical scenarios, choices were not mutually exclusive. In fact, two or more management choices were often possible, such as calling the doctor and undertaking an action (giving the patient oxygen, for example). Therefore, the inclusion of both responses in our analyses allowed us to disregard the sequence in which the actions were performed. Furthermore, when these actions are often accomplished almost simultaneously, choosing only the first response could bias the results.

For each of the theoretical 196 doctor-nurse pairs, there were six scenarios about nurses’ role and five scenarios about doctors’ role (Table 2). We considered the following covariates: prior experience in Internal Medicine was defined as a binary variable with a cut-off at 36 months. We used this cut-off point because it is the duration of postgraduate internal medicine training as an intern in our teaching hospital. The question type was defined by two variables: one was about doctor or nurse roles, and the other about the acuteness of the case. A case was either acute if it required immediate attention, or non-acute: sub acute when needing rapid management (before the planned medical rounds), and not acute when action was required in the next few hours, and when decisions could be addressed during the next rounds. Based on a consensus of three expert clinicians, three of the 11 cases were acute. We also included gender, as the proportion of female nurses was much higher than that of female doctors. We considered prior Emergency Medicine (ER) and Intensive Care (ICU) rotations as potential confounders. Finally, we also included a variable for the country of training to compare training in Switzerland to training in any other country. We did not include overall clinical experience in our model, due to the co linearity between total clinical experience and prior experience in the Division of Internal Medicine, and because internal medicine doctors often have little other clinical experience. Likewise, age was highly correlated to prior experience in Internal Medicine, and therefore was not included in the model.

**Table 2:** Description of clinical cases in the questionnaire.

Clinical scenarios		Acuteness	Description
1	Nursing	Not acute	agitation for suspected robbery
2	Nursing	Acute	chest pain after MI
3	Nursing	Not acute	gout
4	Nursing	Not acute	cutaneous allergic reaction
5	Nursing	Sub acute	dehydration
6	Nursing	Not acute	discontented patient
7	Doctor	Acute	unconscious patient (carbonarcosis)
8	Doctor	Acute	hypoxia
9	Doctor	Sub acute	double insulin dose
10	Doctor	Sub acute	repeated known hemoptysis
11	Doctor	Sub acute	pleural effusion and dyspnea

The concordance outcome measures were correlated. For each doctor-nurse pair, there were 11 correlated responses corresponding to each scenario: a doctor-nurse pair was nested within a doctor and a nurse, but nurses were not nested within doctors or vice versa. Therefore, we had data correlated within multiple levels that were not nested within each other. To account for this correlation between questions and also between doctor-nurse pairs, we used the multistep Generalized Estimating Equations proposed by Miglioretti et al. [24]. The multistep method yields correct standard errors for confidence intervals and Wald hypothesis tests, and concordance estimates have marginal interpretation even when the units of analysis are correlated, so the interpretation does not depend on specific doctor-nurse pairs. Given the nature of our data and analyses, we used a bootstrap method for the power estimation. Because the effect size is quite large (adjusted OR for number of females = 1.32 in our dataset), the estimated power is 90% even if we only have 14 doctors and 14 nurses. We used this approach for the logistic model, comparing unadjusted results, and adjusted results for years of clinical experience in Internal Medicine, type of roles (doctor vs nurse), acuteness of clinical cases, gender, prior rotation in the ER and/or ICU, and whether training took place in Switzerland or not.

## Results

Prior experience in the Division of General Internal Medicine was similar (mean of 3 years for residents, 4 years for the nurses). The average age of participants was 31 years for residents (range 25–36 years), and 37 years for nurses (range 27–48 years). Doctors and nurses differed in gender proportions (male to female ratio 10:4 among doctors, and 4:10 among nurses), and in place of training (ratio of Swiss-trained to training abroad of 10:4 among doctors vs 4:10 among nurses) [22]. Overall, the response rate to the eleven questions was very high. The participants responded to 99% of the questions with at least one choice. They provided a second choice in the majority of cases (66% for doctors and 70% for nurses). For one of the doctor questions, participants only had one choice (only one nurse provided two choices). Agreement among doctors and among nurses increased when considering the top two choices rather than just the first choice (Table 3). The number of choices ranged from one to five. Likewise, analyses of concordance by individual showed some variation, but no one had constantly atypical responses (Tables not shown).

**Table 3:** Proportion of agreement by question when considering one or two top answers, and by type of question (doctor or nurse questions).

Questions (type)		Proportion agreed (top one)	Proportion agreed (top two)
1	Nursing	0.38	0.85
2	Nursing	0.72	1
3	Nursing	0.29	0.71
4	Nursing	0.37	0.66
5	Nursing	0.36	0.56
6	Nursing	0.48	0.68
7	Doctor	0.36	0.51
8	Doctor	0.2	0.51
9	Doctor	0.68	0.68
10	Doctor	0.33	0.56
11	Doctor	0.32	0.39
Overall:			
Doctors		0.42	0.74
Nurses		0.38	0.53

We present in Table 4 the odds ratios of concordance among doctor and nurse pairs using the initial response, in both unadjusted and adjusted analyses. Results were very similar in the unadjusted and adjusted analyses. The only significant association between covariates and concordance was the number of female professionals in the doctor-nurse pairs.

**Table 4:** Odds ratios for doctor-nurse concordance on first professional action in 11 clinical cases of Internal Medicine.

	Unadjusted		Adjusted	
Doctor-nurse pair characteristics:				
Prior Internal Medicine Experience	1.17**	(1.03,1.33)	1.12*	(0.97,1.28)
Number of female professional(s)	1.21***	(1.05,1.39)	1.18***	(1.06,1.32)
Prior ER training	1.11	(0.93,1.32)	1.09	(0.94,1.28)
Prior ICU training	0.99	(0.84,1.17)	0.99	(0.87,1.13)
Swiss training	1.15	(0.90,1.47)	1.16*	(0.96,1.41)
Clinical cases characteristics:				
Doctor scenarios	0.76	(0.52,1.11)	0.77	(0.49,1.22)
Acute scenarios	1.19	(0.80,1.77)	1.11	(0.69,1.78)

*p<0.10,

**p<0.05,

***p<0.01.

Referent groups are: <36 months internal medicine experience, no female professionals, no prior ER training, no prior ICU training, training outside Switzerland, nurse scenarios and not acute scenarios (sub acute or not acute)

Results from the logistic regression model comparing doctor-nurse concordance of the participants’ top two choices for each question is presented in Table 5. Results were similar in the unadjusted and adjusted analyses, other than for the acuteness of cases, which was only significantly associated with concordance in the adjusted analysis. The odds of agreement did not increase with prior experience in a common environment, prior acute care training (ER or ICU) or with the country of training. We found a significant association between the number of female professionals and the concordance between doctor and nurse pairs (adjusted OR 1.32 95%CI 1.02 to 1.73 p < .01), after adjusting for the other covariates. Pairs with two female professionals reached a higher odds of concordance than if there was only one woman in the pair, and that a pair with one female professional reached a higher odds of agreement than if there were two men in the pair. The odds ratio of doctor-nurse concordance was significantly higher in scenarios with acute cases, compared to non-acute cases.

**Table 5:** Odds ratios for doctor-nurse concordance of top two professional actions in 11 clinical cases of Internal Medicine.

	Unadjusted		Adjusted	
Doctor-nurse pair characteristics:				
Prior Internal Medicine Experience	1.26	(0.93,1.56)	1.22	(0.91,1.65)
Number of female professional(s)	1.34**	(1.03,1.75)	1.32**	(1.02,1.73)
Prior ER training	1.01	(0.74,1.38)	0.91	(0.67,1.25)
Prior ICU training	1.15	(0.84,1.56)	1.13	(0.86,1.49)
Swiss training	0.94	(0.70,1.25)	0.86	(0.65,1.15)
Clinical cases characteristics:				
Doctor scenarios	1.84***	(1.17,2.90)	2.19***	(1.32,3.65)
Acute scenarios	1.57*	(0.94,2.65)	2.02***	(1.13,3.62)

*p<0.10,

**p<0.05,

***p<0.01.

Referent groups are: <36 months internal medicine experience, no female professionals, no prior ER training, no prior ICU training, training outside Switzerland, nurse scenarios and not acute scenarios (sub acute or not acute)

## Discussion

Sharing a similar team mental model of both doctor and nurse roles is an important basis for interprofessional collaboration, and some factors may predict a higher doctor-nurse concordance of roles and expectations in the initial management of some clinical scenarios. In our study, the odds of concordance between doctors and nurses on expected actions was associated with the number of female professionals in doctor-nurse pairs, and even after adjusting for prior experience in internal medicine or acute medicine, place of training and type of questions. This factor also had similar influence in analyses using either first choice or first two-choices of actions. Based on participants’ initial responses to our set of clinical scenarios (top two-choice model), the concordance between doctors and nurses was higher for doctor role questions and for acute case management questions in the multivariate analyses. Although prior experience in Internal Medicine was a significant factor in the top choice model, our results did not consistently confirm this hypothesis in the top two-choice model. Our hypothesis for acute medicine and about country of training being associated with higher odds of agreement between doctors and nurses were also not confirmed. These results suggest that adaptation to the habits of the current professional environment may erase differences with habits originating from the past professional curriculum and working environment. This is in line with the "hidden curriculum" perspective [25].

The findings about the correlation with doctor or nurse scenarios support our previous results on the roles of doctors and nurses [22]. In that study, we attributed the initial findings of better correlation for nurses’ scenarios than for doctors’ scenarios to the weaker correlation on the item regarding nurses’ autonomy. In the qualitative results, nurses considered themselves more autonomous than was perceived by the doctors [22]. This tendency is also suggested in the present study when analyzing the first response to the questionnaire (Table 4). Nurses usually handle the initial responses to patient needs, often prior to the arrival of the doctor at the bedside. In our present analyses, we focused on the expectations for the immediate, initial actions of doctors and nurses to a variety of clinical situations, rather than on the overall approach and role of each professional, to get a better notion of role perceptions and expectations. When considering the top two choices for management, doctor management strategies had significantly higher odds of agreement than nursing management strategies in the given clinical situations. This finding suggests that doctor and nurses may have different notions of the order in which tasks need to be performed, even though they may agree on the overall content. Furthermore, tasks may also be performed simultaneously, particularly in acute or urgent situations. This finding suggests that our choice of comparing the two first actions was relevant to actual practice.

Concordance in doctor role and expectations may be affected by differences in clinical approaches of doctors and nurses. When the doctor arrives at the bedside, he/she takes on the role of decision-maker with a more abstract approach. While a nurse may anticipate administering oxygen if a patient’s oxygen saturations drop, a doctor will probably also evaluate the need for ventilation, the type of radiology work-up, blood tests and other procedures, addressing not only the immediate problem, but also determining the etiology of the clinical problem and management plan. Low concordance could also be due to a difference in case management (i.e., what actions are needed) or in case perception (degree of severity or urgency) [26].

The management of acute case scenarios seems significantly associated with better doctor-nurse concordance in the present study. This finding underlines the doctors’ dependence on nurses to initiate assessment and actions at the bedside in response to unexpected events. Acute care situations often trigger life-supporting response tasks, and often have a clearly defined protocol. It is therefore not surprising that the acute cases reach higher odds for interprofessional agreement. The implications of this finding are important and should guide interprofessional education towards establishing clearer guidelines for mutual expectations of responses to less acute situations.

Gender differences are known to affect professional relationships, particularly among different professions like doctors and nurses [27]. The effect of female gender on concordance found in both the top choice model and top two choices model suggests that two women were more likely to agree on expected nurses’ and doctors’ actions than if one or both were men. The stratified analyses show an increasing trend of concordance from male-male (61% agreement on the first two choices), mixed gender (68% agreement) to female-female (74% agreement). These results reflect findings from prior literature on teamwork and gender in medical and non-medical contexts [27–30]. Gender differences seem to start before clinical work as female nurses and medical students have better attitudes toward interprofessional collaboration [31–34]. Differences in cognitive abilities such as attention [35, 36] and approach to solving complex problems in risky situations [37] may explain these results. Other hypotheses include better reliance of female physicians on clinical guidelines and evidence-based practice and more patient-centered care [38]. Gender effect and its causes is a particularly interesting issue for future interprofessional collaboration, given a growing number of women among the doctor workforce, and a predominantly female nurse workforce.

An increasing number of healthcare professionals in Switzerland are trained in other countries: the proportion of doctors trained outside of Switzerland increased from 29% to 35% between 2002 and 2008, and the proportion of nurses from 35% to 38%; the proportion of French-trained healthcare professionals working in Swiss hospitals increased from 33% to 36% during this period [39]. This issue is particularly relevant for the Geneva University Hospitals, with its high demand for nursing staff and close proximity to France. Contrary to our initial hypothesis, the country of training was not significantly associated with similarity in team mental model. We suspect that any potential effect of country of training, may decrease with time and experience gained in Internal Medicine. Testing our hypothesis adequately would require a larger sample, which would allow us to compare responses and expectations of newly trained doctors and nurses with that of more experienced professionals. We also cannot exclude that our dataset may simply be too small to show a statistically significant effect.

### Strength and limitations

Choosing to create a dataset with a concordance variable that takes into consideration any combination of doctor and nurse is consistent with the reality, where the occurrence of doctors teaming up with different nurses (and vice versa) is common, and it allows us to somewhat improve the generalizability of results. Another strength of our study is that our analyses allow us to take into consideration personal characteristics and other potential confounders in a model for concordance among doctors and nurses.

Our 11-case questionnaire was created based on commonly encountered situations in internal medicine, which were familiar to all participants. Our questions should therefore allow for reasonable generalizability of results in terms of case specificity. Limitations of our study include the inclusion of only two health professionals in the study population. This choice is partly justified by our local practices, where other professions (physiotherapy, dietician, pharmacists, etc.) are not systematically involved in all areas of patient care. Using clinical scenarios addresses some facets of mental models but does not allow for a complete assessment. Future research should strive to develop ways to perform even more complete assessment. Furthermore, lack of concordance may arise from differences in patient management between doctors and nurses (i.e., nurses and doctors are trained to take care of different aspects of a given clinical situation) rather than from different team mental models. Using the method described in this paper, we intend to conduct a larger study to confirm the results of our analyses, and to further explore the role of gender in doctor-nurse concordance. Having a better understanding of the current state of interprofessional collaboration is a key component to developing tailored interventions for future interprofessional education. Finally, as underlined in our results, expectations and role perceptions are highly influenced by environmental conditions. This implies that our findings may not be generalizable to other medical specialties (surgery, pediatrics, for example), nor for other medical settings (different countries, for example). Future studies are needed to study cultural differences for interprofessional collaboration in these other specialties.

## Conclusion

Identifying factors that affect interprofessional team mental models, in particular role perceptions and expectations between doctors and nurses can help guide the development and implementation of educational interventions aimed at improving interprofessional collaboration. In our study, concordance between doctors and nurses was lower for non-acute situations, and for doctor roles. Our brief questionnaire-based approach and concordance-based analyses may represent a simple, feasible method, which can be adapted to include more than two types of professionals, to rapidly assess the similarity of team mental models.

## Supporting information

S1 Dataset The questionnaire dataset. (XLSX)

## References

[1] Verschuren PJ, Masselink H. Role concepts and expectations of physicians and nurses in hospitals. Soc Sci Med. 1997;45(7):1135–8. 9257405 10.1016/s0277-9536(97)00043-9

[2] Gordon MB, Melvin P, Graham D, Fifer E, Chiang VW, Sectish TC, et al Unit-based care teams and the frequency and quality of physician-nurse communications. Archives of pediatrics & adolescent medicine. 2011;165(5):424–8. 21536957 10.1001/archpediatrics.2011.54

[3] Martin JS, Ummenhofer W, Manser T, Spirig R. Interprofessional collaboration among nurses and physicians: making a difference in patient outcome. Swiss medical weekly. 2010;140:w13062 doi: 10.4414/smw.2010.13062 20458647 10.4414/smw.2010.13062

[4] Zwarenstein M, Goldman J, Reeves S. Interprofessional collaboration: effects of practice-based interventions on professional practice and healthcare outcomes. Cochrane Database Syst Rev. 2009(3):CD000072. 10.1002/14651858.CD000072.pub2 19588316

[5] Ferguson SL. TeamSTEPPS: integrating teamwork principles into adult health/medical-surgical practice. Medsurg nursing: official journal of the Academy of Medical-Surgical Nurses. 2008;17(2):122–5. 18517173

[6] Baggs JG, Schmitt MH. Nurses' and resident physicians' perceptions of the process of collaboration in an MICU. Research in nursing & health. 1997;20(1):71–80. 9024479 10.1002/(sici)1098-240x(199702)20:1<71::aid-nur8>3.0.co;2-r

[7] Curley C, McEachern JE, Speroff T. A firm trial of interdisciplinary rounds on the inpatient medical wards: an intervention designed using continuous quality improvement. Medical care. 1998;36(8 Suppl):AS4–12. 9708578 10.1097/00005650-199808001-00002

[8] Klimosky R M S. Team mental model: construct or metaphor. Journal of Management. 1994;20(2):403–37.

[9] Mathieu JE, Heffner TS, Goodwin GF, Salas E, Cannon-Bowers JA. The influence of shared mental models on team process and performance. J Appl Psychol. 2000;85(2):273–83. 10783543 10.1037/0021-9010.85.2.273

[10] DeChurch LA, Mesmer-Magnus JR. Measuring shared team mental models: A meta-analysis. Educational Publishing Foundation; 2010.

[11] Ensley MD, Pearce CL. Shared cognition in top management teams: implications for new venture performance. J Organ Behav. 2001;22:145–60.

[12] Ebert L, Hoffman K, Levett-Jones T, Gilligan C. "They have no idea of what we do or what we know": Australian graduates' perceptions of working in a health care team. Nurse education in practice. 2014;14(5):544–50. doi: 10.1016/j.nepr.2014.06.005 24999074 10.1016/j.nepr.2014.06.005

[13] Powell AE, Davies HT. The struggle to improve patient care in the face of professional boundaries. Soc Sci Med. 2012;75(5):807–14. doi: 10.1016/j.socscimed.2012.03.049 22633159 10.1016/j.socscimed.2012.03.049

[14] Manser T. Teamwork and patient safety in dynamic domains of healthcare: a review of the literature. Acta anaesthesiologica Scandinavica. 2009;53(2):143–51. doi: 10.1111/j.1399-6576.2008.01717.x 19032571 10.1111/j.1399-6576.2008.01717.x

[15] Mazzocco K, Petitti DB, Fong KT, Bonacum D, Brookey J, Graham S, et al Surgical team behaviors and patient outcomes. American journal of surgery. 2009;197(5):678–85. doi: 10.1016/j.amjsurg.2008.03.002 18789425 10.1016/j.amjsurg.2008.03.002

[16] Tang CJ, Chan SW, Zhou WT, Liaw SY. Collaboration between hospital physicians and nurses: an integrated literature review. International nursing review. 2013;60(3):291–302. doi: 10.1111/inr.12034 23961790 10.1111/inr.12034

[17] Weller JM, Janssen AL, Merry AF, Robinson B. Interdisciplinary team interactions: a qualitative study of perceptions of team function in simulated anaesthesia crises. Medical education. 2008;42(4):382–8. doi: 10.1111/j.1365-2923.2007.02971.x 18338990 10.1111/j.1365-2923.2007.02971.x

[18] Sawatzky JA, Enns CL. Exploring the key predictors of retention in emergency nurses. Journal of nursing management. 2012;20(5):696–707. doi: 10.1111/j.1365-2834.2012.01355.x 22823226 10.1111/j.1365-2834.2012.01355.x

[19] Van Bogaert P, Meulemans H, Clarke S, Vermeyen K, Van de Heyning P. Hospital nurse practice environment, burnout, job outcomes and quality of care: test of a structural equation model. Journal of advanced nursing. 2009;65(10):2175–85. 20568322 10.1111/j.1365-2648.2009.05082.x

[20] Abu-Rish E, Kim S, Choe L, Varpio L, Malik E, White AA, et al Current trends in interprofessional education of health sciences students: a literature review. Journal of interprofessional care. 2012;26(6):444–51. doi: 10.3109/13561820.2012.715604 22924872 10.3109/13561820.2012.715604 PMC7594101

[21] Kitto S, Goldman J, Schmitt MH, Olson CA. Examining the intersections between continuing education, interprofessional education and workplace learning. Journal of interprofessional care. 2014;28(3):183–5. doi: 10.3109/13561820.2014.906737 24702045 10.3109/13561820.2014.906737

[22] Muller-Juge V, Cullati S, Blondon KS, Hudelson P, Maitre F, Vu NV, et al Interprofessional collaboration on an internal medicine ward: role perceptions and expectations among nurses and residents. PloS one. 2013;8(2):e57570 doi: 10.1371/journal.pone.0057570 23469027 10.1371/journal.pone.0057570 PMC3585159

[23] McNeese NRMFE. Towards a team mental model of collaborative information seeking during team decision-making. Proceedings of the Human Factors and Ergonomics Society Annual Meeting. 2014;58(1):335–9.

[24] Miglioretti DL, Heagerty PJ. Marginal modeling of nonnested multilevel data using standard software. American journal of epidemiology. 2007;165(4):453–63. doi: 10.1093/aje/kwk020 17121864 10.1093/aje/kwk020

[25] Hafler JP, Ownby AR, Thompson BM, Fasser CE, Grigsby K, Haidet P, et al Decoding the learning environment of medical education: a hidden curriculum perspective for faculty development. Academic medicine: journal of the Association of American Medical Colleges. 2011;86(4):440–4. 21346498 10.1097/ACM.0b013e31820df8e2

[26] Neville TH, Wiley JF, Yamamoto MC, Flitcraft M, Anderson B, Curtis JR, et al Concordance of Nurses and Physicians on Whether Critical Care Patients are Receiving Futile Treatment. American journal of critical care: an official publication, American Association of Critical-Care Nurses. 2015;24(5):403–10. 10.4037/ajcc2015476 26330433

[27] Zelek B, Phillips SP. Gender and power: Nurses and doctors in Canada. International journal for equity in health. 2003;2(1):1 doi: 10.1186/1475-9276-2-1 12605720 10.1186/1475-9276-2-1 PMC150379

[28] Ivanova-Stenzel R, Kübler D. Gender differences in team work and team competition. Journal of Economic Psychology. 2011;32(5):797–808.

[29] Hall P. Interprofessional teamwork: professional cultures as barriers. Journal of interprofessional care. 2005;19 Suppl 1:188–96. 16096155 10.1080/13561820500081745

[30] Molina JA, Gimenez-Nadal JI, Cuesta JA, Gracia-Lazaro C, Moreno Y, Sanchez A. Gender Differences in Cooperation: Experimental Evidence on High School Students. PloS one. 2013;8(12). 10.1371/journal.pone.0083700 PMC3867463 24367608

[31] Wilhelmsson M, Ponzer S, Dahlgren LO, Timpka T, Faresjo T. Are female students in general and nursing students more ready for teamwork and interprofessional collaboration in healthcare? BMC Med Educ. 2011;11:15 doi: 10.1186/1472-6920-11-15 21510872 10.1186/1472-6920-11-15 PMC3110123

[32] Lindh Falk A, Hammar M, Nystrom S. Does gender matter? Differences between students at an interprofessional training ward. Journal of interprofessional care. 2015;29(6):616–21. doi: 10.3109/13561820.2015.1047491 26652634 10.3109/13561820.2015.1047491

[33] Hansson A, Foldevi M, Mattsson B. Medical students' attitudes toward collaboration between doctors and nurses—a comparison between two Swedish universities. Journal of interprofessional care. 2010;24(3):242–50. doi: 10.3109/13561820903163439 19995272 10.3109/13561820903163439

[34] Reynolds F. Initial experiences of interprofessional problem-based learning: a comparison of male and female students' views. Journal of interprofessional care. 2003;17(1):35–44. doi: 10.1080/1356182021000044148 12772468 10.1080/1356182021000044148

[35] Feng Q, Zheng Y Fau—Zhang X, Zhang X Fau—Song Y, Song Y Fau—Luo Y-J, Luo Yj Fau—Li Y, Li Y Fau—Talhelm T, et al. Gender differences in visual reflexive attention shifting: evidence from an ERP study. (1872–6240 (Electronic)). 10.1016/j.brainres.2011.05.041 21663895

[36] Jausovec N, Jausovec K. Gender related differences in visual and auditory processing of verbal and figural tasks. (1872–6240 (Electronic)). 10.1016/j.brainres.2009.08.093 19747461

[37] Charness G, Gneezy U. Strong Evidence for Gender Differences in Risk Taking. Journal of Economic Behavior & Organization. 2012;83(1):50–8.

[38] Baumhakel M, Muller U Fau—Bohm M, Bohm M. Influence of gender of physicians and patients on guideline-recommended treatment of chronic heart failure in a cross-sectional study. (1388–9842 (Print)). 10.1093/eurjhf/hfn041 PMC2645055 19158153

[39] Jaccard Ruedin H, Widmer M. L’immigration du personnel de santeé vers la Suisse. 2010.

